# Automated evaluation of probe-based confocal laser endomicroscopy in the lung

**DOI:** 10.1371/journal.pone.0232847

**Published:** 2020-05-06

**Authors:** David Bondesson, Moritz J. Schneider, Edith Silbernagel, Jürgen Behr, Frank Reichenberger, Julien Dinkel

**Affiliations:** 1 Department of Radiology, University Hospital, LMU Munich, Munich, Germany; 2 Comprehensive Pneumology Center (CPC-M), University Hospital, LMU Munich, Helmholtz Zentrum München, Member of the German Center for Lung Research (DZL), Munich, Germany; 3 Department of Pneumology, Asklepios Fachklinikun Munich-Gauting, Member of the German Center for Lung Research (DZL), Munich, Germany; 4 Department of Internal Medicine V, University of Munich (LMU), Munich, Germany; 5 Department of Radiology, Asklepios Lung Center Munich-Gauting, Munich, Germany; University of Houston, UNITED STATES

## Abstract

**Rationale:**

Probe-based confocal endomicroscopy provides real time videos of autoflourescent elastin structures within the alveoli. With it, multiple changes in the elastin structure due to different diffuse parenchymal lung diseases have previously been described. However, these evaluations have mainly relied on qualitative evaluation by the examiner and manually selected parts post-examination.

**Objectives:**

To develop a fully automatic method for quantifying structural properties of the imaged alveoli elastin and to perform a preliminary assessment of their diagnostic potential.

**Methods:**

46 patients underwent probe-based confocal endomicroscopy, of which 38 were divided into 4 groups categorizing different diffuse parenchymal lung diseases. 8 patients were imaged in representative healthy lung areas and used as control group. Alveolar elastin structures were automatically segmented with a trained machine learning algorithm and subsequently evaluated with two methods developed for quantifying the local thickness and structural connectivity.

**Measurements and main results:**

The automatic segmentation algorithm performed generally well and all 4 patient groups showed statistically significant differences with median elastin thickness, standard deviation of thickness and connectivity compared to the control group.

**Conclusion:**

Alveoli elastin structures can be quantified based on their structural connectivity and thickness statistics with a fully-automated algorithm and initial results highlight its potential for distinguishing parenchymal lung diseases from normal alveoli.

## Introduction

Diagnostics of diffuse parenchymal lung disease (DPLD) is to this day a complex task performed using the collective information from clinical, radiological and histological criteria and analysed in a multidisciplinary discussion [1–3].

However, even gold standard imaging methods such as high resolution computer tomography (HRCT) [4,5] struggle to yield consensus with regards to diagnosis in DPLD with a large interreader variability [5], advocating a need for additional diagnostic information in equivocal cases [6]. For this reason, pathological specimens are sometimes necessary in the diagnostic workup, such as surgical lung biopsy and more recently bronchoscopic cryobiopsy [7]. However, invasive tissue sampling methods, risk causing complications such as pneumothorax, haemorrhage and acute exacerbation of the DPLD. To address this issue, probe-based confocal laser endomicroscopy (pCLE) has been presented as a novel technique for providing imaging of the respiratory tract and alveolar ducts in real time [8] based on the microstructures’ autofluorescence [9].

Image acquisition is done by introducing the pCLE probe through a flexible bronchoscope during standard examination. The probe captures 12 images/second with the following image parameters: Distal diameter = 1.4 *mm*, Field of view = 600 *μm*, imaging depth = 0 − 50 *μm*, lateral resolution = 3.5 *μm*, axial resolution = 15 *μm*. The probe diameter of 1.4 mm ensures that it can be pushed deep into the lung. It emits laser light with a wavelength of 488 nm which excites autofluorescence from the elastin content in the alveoli structures. With a multitude of technical improvements over the last 15 years to overcome the low specificity of autofluorescence defects [10,11], pCLE has shown promise as a diagnostic method to visualize lung tissue in vivo [12,13]. Multiple studies [14–17] have investigated the structural changes of lung tissue caused by different DPLDs and specifically highlighted increased elastin fibre thickness, density of fibres and number of cellular structures as important features. The aim of this study was to develop a fully automatic workflow for quantifying these structural properties using pCLE measurements and to perform a preliminary assessment of their diagnostic potential.

## Materials and methods

### Patient characteristics

46 patients were included in this study (mean age±standard deviation = 70.1 ± 8.2, 30 male and 16 female, 29 ex-smokers (since more than 10 years), 15 non-smokers and 2 without info). All patients were newly diagnosed in accordance with histological, radiological and clinical results based on a multidisciplinary discussion. Each patient was assessed according to current guidelines including HRCT, biopsy, pulmonary function test with blood gas analysis and 6-minute-walk test. All were referred for examination as part of workup of newly diagnosed DPLD and in stable clinical condition. Of these, 11 were diagnosed with cryptogenic organizing pneumonia (COP), 8 with non-specific interstitial pneumonia (NSIP), 11 with idiopathic pulmonary fibrosis (IPF) and 8 with hypersensitive pneumonia (HP). 5 patients with Sarcoidosis (without histological lung pulmonary involvement) were classified as normal parenchyma. Additionally, pCLE was performed on the contralateral healthy lung of one patient with an allergic bronchopulmonary aspergillosis as well as one with bronchial pneumonia. HRCT showed no abnormality in these unaffected lungs. Lastly, pCLE was performed on a patient with metastases from breast cancer in unaffected parts of the lung. Altogether, this made for 8 patients classified as having normal elastin structure. Exclusion occurred based on severe restriction (vital capacity or total lung capacity below 50% pred.), severe hypoxaemia (pO2 < 55 mmHg), congenital or acquired disorder of the coagulation system, signs of pulmonary hypertension, signs of infection, exacerbation or inability to undergo bronchoscopy for any medical or legal reason. The study was approved by the local ethics committee of the Ludwig Maximilians University Munich, Germany, (Record number 048/13). All patients obtained information by a pulmonologist and gave their written informed consent to use the pCLE mini probe during the bronchoscopy 24 hours prior to examination.

### Image acquisition

**Fig 1:**
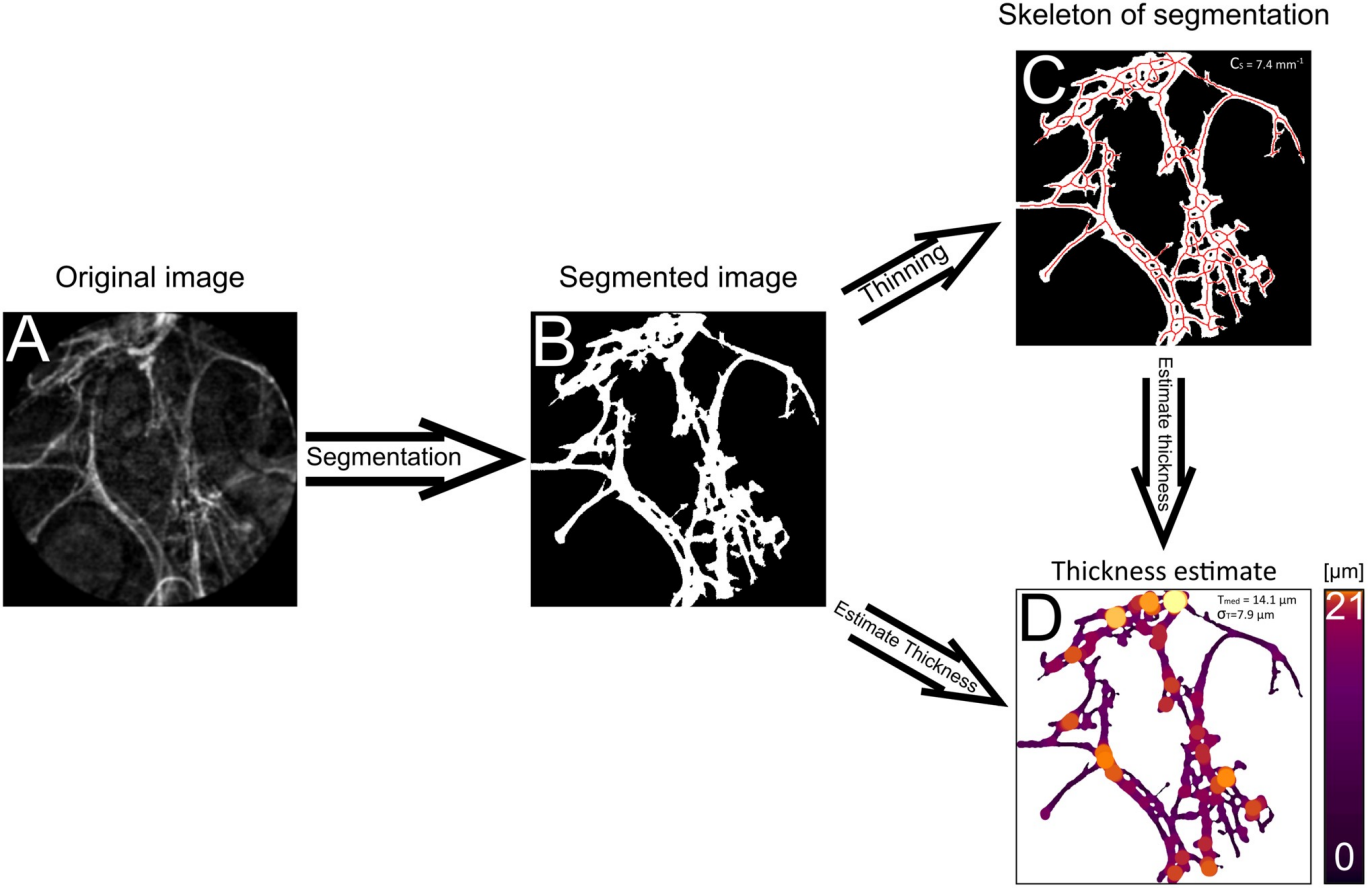
Workflow for structural evaluation of alveoli elastin. A) Example of acquired NSIP patient snapshot from pCLE screening. B) Resulting segmentation generated by pixel classification based on a machine learning approach. C) Generated skeleton of structure used when calculating local thickness. D) Visualization of local thickness for whole structure.

The patients underwent bronchoscopy examination in combination with pCLE (Cellvizio, Mauna Kea Technologies, France, Paris) with varying duration. The bronchoscopy examination was performed according to guidelines in rigid technique with patients under general anaesthetic using a flexible bronchoscope (BF-Q 180, Olympus, Japan) to collect mucus samples for microbiological and cytological assessment as well as examine the bronchial tree. Next, the pCLE probe was pushed through the working channel of the bronchoscope and further into the peripheral compartment of the bronchial tree with fluoroscopic guidance until elastin fibres of alveoli ducts were reached. PCLE was performed in regions of the lung which showed clear signs of pathology from the HRCT images. Neighbouring sub-segments were also examined in cases where no obvious pathological structure could be observed. No exogenous fluorophores were required for this procedure. The pCLE recordings were taken during extractive motion of the probe to limit contact pressure of the probe onto the tissue surface. Using Cellvizio Viewer Software v.1.6.0 (Mauna Kea Technologies, Paris, France), snapshots from the recordings that displayed characteristic alveoli elastin structure without elastin tension or procedure related changes were selected by two pulmonologist experienced in interventional rigid bronchoscopy to be quantified (Fig 1A). The pulmonologists were unaware of the results of other performed diagnostic tests.

### Image processing

A machine learning model [18] was trained for the fully automatic segmentation. Model training and pixel classification were performed in ‘Trainable Weka Segmentation’ [19] called from ImageJ (Fiji) [20] as a plugin. 23 snapshots were manually labelled by the pulmonologists (resulting in 267 elastin areas and 232 background areas constituting a total of 1060543-pixel instances). The snapshots were randomly selected resulting in a set of 4 NSIP cases, 15 IPF cases and 2 normal cases. 21 of the snapshots were used for training and tuning the classifier. Since there were approximately 2.5 times more instances of background than elastin structure in the labels, random undersampling [21] was performed to rebalance the classes. Features were generated from a multitude of image filtering methods available in Trainable Weka Segmentation that extract different spatial characteristics from an image. Classifiers were trained on instances with an added correlation-based feature selection step [22] to minimize their intraclass correlation while improving upon prediction accuracy. Features were ranked based on their information gain ratio. The last 2 snapshots (with 77650 instances) were used as a test set for comparing the classifiers. A random forest classifier with 200 trees yielded highest receiver operating characteristics on the test images (area of 98.8% under the curve) and was thus selected for segmentation. A total of 131 representative screenshots from the 46 patients were segmented (Fig 1B) using this model. Representative shots entailed those that showed characteristic elastin structure for pathological or normal healthy structure in accordance with described criteria [14]. The segments were subsequently visually assessed by the two pulmonologists to ensure that elastin structures of interest were included, and background textures removed. In some instances, pixel classification was not able to determine with certainty if some regions were holes or structure and could generate small regions that looked like pixel noise with a mixture of both. To prevent an artificial increase of holes, those with a distance between each other smaller than 2 *μm* were merged and holes smaller than 30 *μm* filled in. Numerical values were chosen empirically by pulmonologists. Due to an imbalanced number of snapshots per patient, only the first snapshot from each examination was processed and used for the statistical evaluation. Due to low signal to noise ratio (SNR) in some acquired snapshots leading to segmentation irregularities, one HP measurement was excluded and in two measurements the second snapshot was instead used for evaluation. The segments were then evaluated based on their structural tissue connectivity *C*_*s*_ (Fig 1C), median and standard deviation of local thickness (*T*_med_ and *σ*_*T*_, respectively) (Fig 1D).

#### Image processing—Connectivity calculation

*C*_*s*_ aimed to detect increases of intricacies in the elastin structure associated with DPLDs by quantifying the number of holes of the segmented elastin structure normalized with respect to the structure’s size. Firstly, to estimate a structure’s size, a skeleton was generated by applying a thinning algorithm (skeletonization) [23], creating a one pixel thick topology-preserved medial axis structure (Fig 1C). The skeleton was considered a better value to scale with compared to the binary segmented area, since this was not affected by pixelated edge effects. The number of holes was calculated from the Euler number of the binary snapshots [23]. This quantifies the number of structures and the amount of holes these structures inhibit. *C*_*s*_ was then generated from the number of holes normalized by the total length of the skeleton *L*_*skeleton*_.

#### Image processing—Local thickness estimation

To estimate the local thickness *T*_*local*_ of a structure, the distance from the local centre of the structure to the closest edge was used. Since the generated skeleton represents local midpoint estimations, only the distance to the closest neighbouring pixel *d*_*np*_ for all skeleton points must be determined. The nearest neighbour pixel was found with a k-nearest neighbour algorithm [24] where the pixel with minimum Euclidean distance was selected. *T*_*local*_ was then calculated by doubling *d*_*np*_. Pathology groups were then compared against the characteristically normal group for each parameter resulting in 12 comparisons.

### Statistical evaluation

For statistical comparison the Wilcoxon rank sum test (two-tailed) [25] was used. Differences were considered to be statistically significant for p-values of less than 0.05 after applying Bonferroni correction [26] (number of tests = 12).

## Results

### Image processing

**Fig 2:**
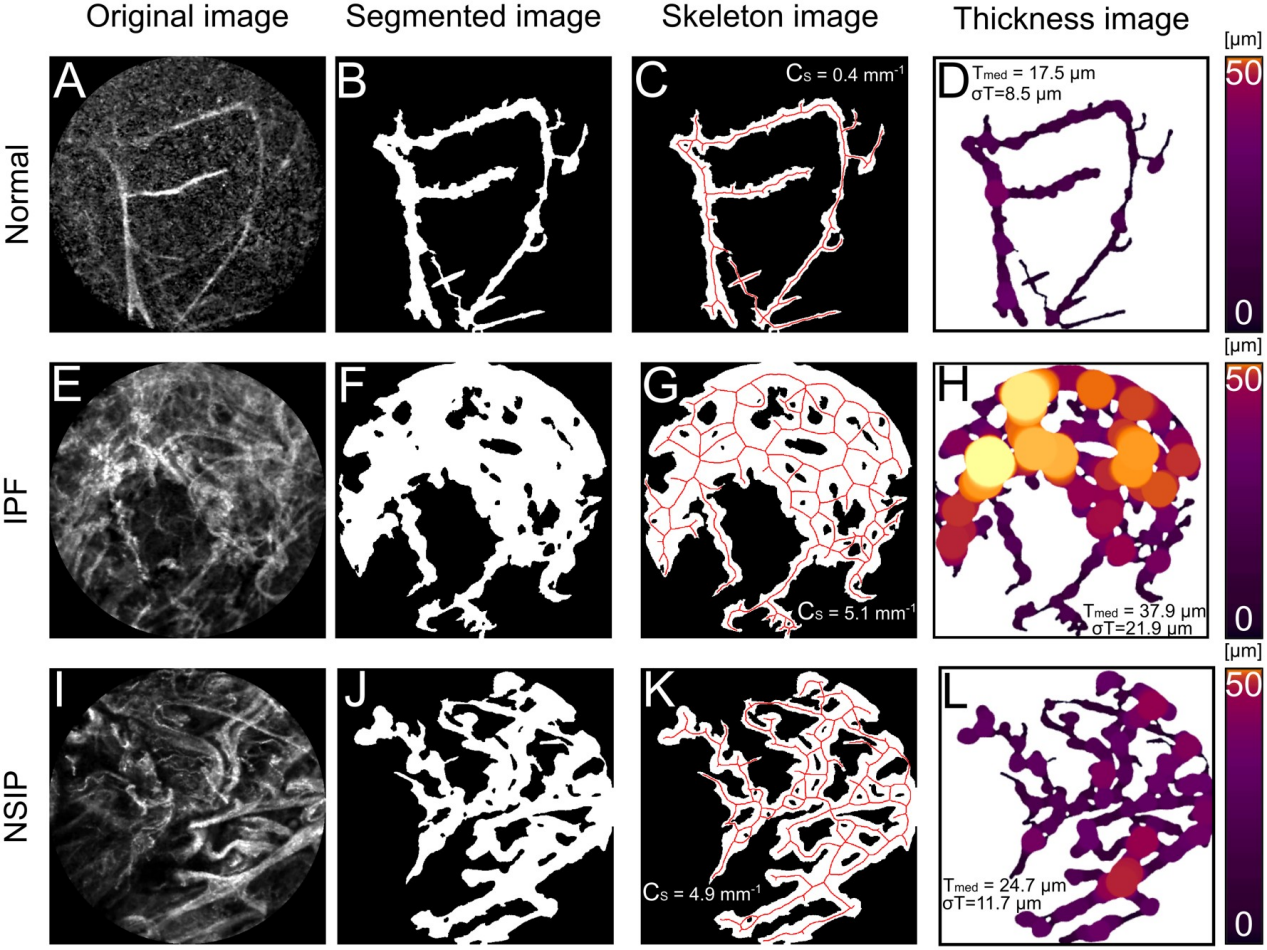
Three examples from every step of evaluation workflow. A-D) represent normal alveoli elastin structure with **T**_**med**_ **= 17.5 μm**, **σ**_**T**_ **= 8.5 μm** and **C**_**s**_ **= 0.4 mm**^**−1**^, E-H) represents IPF elastin structure with **T**_**med**_ **= 37.8 μm, σ**_**T**_ **= 21.9 μm** and **C**_**s**_ **= 5.1 mm**^**−1**^. I-L) represents NSIP structure with **T**_**med**_ **= 24.7 μm, σ**_**T**_ **= 11.7** and **C**_**s**_ **= 4.9 mm**^**−1**^.

The automatic segmentation algorithm performed well on the patient data in accordance with the pulmonologists’ inspection. One HP-patient’s snapshots were removed from the evaluation as only small parts of the structure remained after segmentation. Fig 2 depicts three examples of results generated by the alveoli structure evaluation. Fig 2A displays a normal alveoli structure imaged in low SNR with resulting values *T*_med_ = 17.2 *μm*, *σ*_*T*_ = 8.6 *μm* and *C*_*s*_ = 0.4 *mm*^−1^. Fig 2E displays an IPF patient with characteristic distortion and increased intricacies of the alveolar structure. This resulted in higher values than in the normal tissue example: *T*_med_ = 37.4 *μm*, *σ*_*T*_ = 21.4 *μm* and *C*_*s*_ = 5.2 *mm*^−1^. Fig 2I displays an NSIP patient where an apparent large density was observed. The segmented image distinguished the characteristic crystalline coating and included that as a structure to be evaluated. This too resulted in higher calculated values compared to the normal case: *T*_med_ = 24.3 *μm*, *σ*_*T*_ = 11.6 *μm* and *C*_*s*_ = 4.9 *mm*^−1^.

### Statistical evaluation

**Fig 3:**
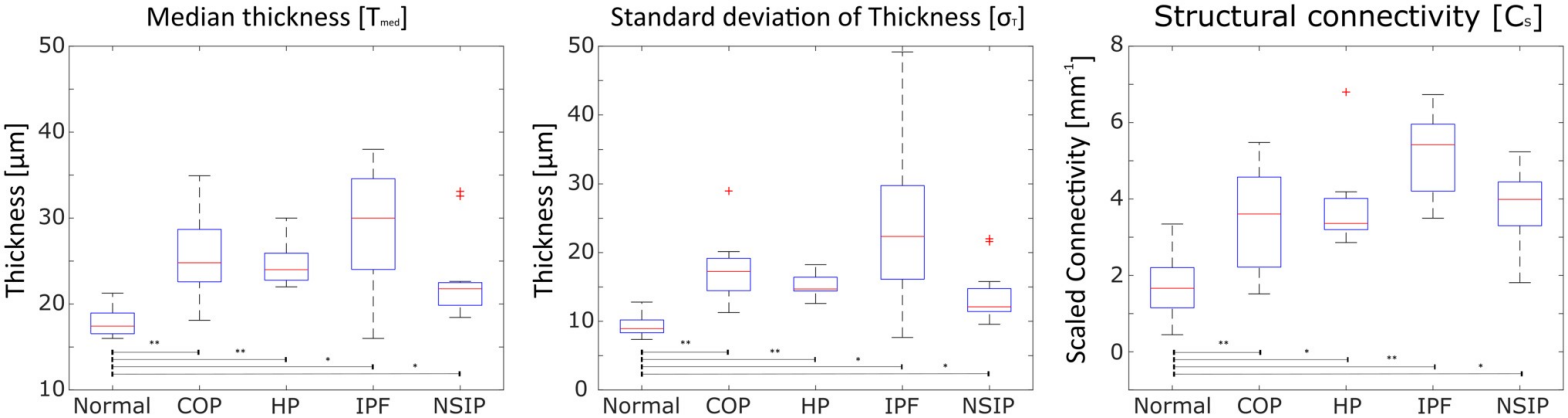
Value distributions of DPLD and normal structure evaluations for structural connectivity and thickness statistics. Box-and-whisker plots displaying value ranges for **T**_**med**_, σ_**T**_ and **C**_**s**_. Red line specifies group median. Bottom and top edges indicate the 25th and 75th percentiles, respectively and whiskers covers **± 2.698σ**. *: p-value<0.05, **: p-value<0.01.

Significant differences were found when comparing the pathological cases against the normal in all of values (Fig 3). When comparing group median of all variables, COP-, HP-, NSIP and IPF- measurements all showed significant increases for *T*_med_, *σ*_*T*_ and *C*_*s*_ with p<0.05.

Table 1 summarizes the group median difference of the three values between all pathology snapshots and the normal snapshots. *T*_med_ differences varied between 4.3 − 12.6 *μm* (*p* < 0.05). *σ*_*T*_ differences varied between 5.8 − 1.4 *μm* (*p* < 0.05). *C*_*s*_ differences varied between 1.70 − 3.76 *mm*^−1^(*p* < 0.05).

**Table 1:** Calculated median values for all measurement groups and difference between DPLDs and normal.

	T_med−_avg[*μ*m]	*σ*_T−avg_[*μ* m]	C_s−avg_[mm^−1^]	ΔT_med−_avg[*μ* m]	Δ*σ*_T−avg_[*μ* m]	ΔC_s−avg_[mm^−1^]
**Normal**	17.4	8.9	1.66	N/A	N/A	N/A
**COP**	24.8	17.2	3.61	7.4 (p = 6.1 * 10^−3^)	8.3 (p = 5.4 * 10^−3^)	1.95 (p = 9.3 * 10^−3^)
**HP**	24.0	14.7	3.36	6.6 (p = 3.7 * 10^−3^)	5.8 (p = 7.5 * 10^−3^)	1.70 (p = 0.013)
**IPF**	30.0	22.3	5.42	12.6 (p = 0.016)	13.4 (p = 0.014)	3.76 (p = 0.3 * 10^−3^)
**NSIP**	21.8	12.1	3.78	4.3 (p = 0.015)	3.2 (p = 0.019)	2.12 (p = 0.013)

## Discussion

We are presenting a follow up study to a previous review article [27], that first suggested a semi-automatic method for the structural evaluation of alveoli elastin. Comparatively, this work presents a fully automatic workflow and demonstrated that the evaluation of elastin structure from pCLE snapshots can distinguish significant differences in elastin thickness and tissue connectivity between normal and DPLD alveoli. When evaluating the structural connectivity, median thickness and standard deviation of thickness COP, HP, NSIP and IPF showed significant differences in all three values compared to normal elastin structure. This suggests that the presented method for quantifying elastin of the alveoli can differentiate normal from diseased structures and can support fully automatic assistance in diagnosis of DPLD. Furthermore, since the method offers multiple parameters for differentiating between normal and pathological groups, more complex methods could potentially be developed for a holistic characterization.

In the presented study we opted for the conservative [28] Bonferroni p-value correction due to a small patient sample size. Since the scope of this study was to show that the presented methods can be of interest as a support tool when looking for DPLDs, we left further validation to future studies with larger sample sizes.

The presented method aims to quantify features that have previously been reported to change due to different DPLDs, such as increase/decrease of elastic fibres, disorganization of elastic network and enlarged axial elastic fibre bundle diameter [14–17]. Similar elastin thickness values were found here as in other work [29], albeit with a higher variability. This is to be expected considering that the presented method takes all of the structure in the FOV into consideration instead of only manually selected parts. To date, we know of no other quantitative analyses for the intricacies of a structure such as the presented structural connectivity value. Other observed structural changes [30] such as alveolar mouth size and increase of fluorescent were not taken into consideration in the presented methods.

Previous studies have focused on expert observer evaluation [14] or manual post processing methods [30,31] when comparing different elastin of the alveoli. The presented method instead offers quantitative values where the whole structure inside the FOV can be evaluated. Using the presented method when evaluating local thickness for example, offers an average of 2734 ± 1278 measurement points per patient which greatly reduces variability from outliers compared to manual thickness estimates.

A machine learning approach was used for the pixel classification to fully automate the segmentation step of the workflow, based on 21 snapshots from all patient measurements. Despite the small number, the segmentation algorithm succeeded in separating structures in noisy snapshots (Fig 2A and 2B) while still ignoring background structures (Fig 2I and 2J). Since DPLD has shown to decrease elastin’s autofluorescence [13,32] it is of high interest to ensure that structures can be extracted even in low SNR snapshots. With the whole workflow running automatically, the opportunity emerges for quantitative assistance in real time during bronchoscopy examination. The presented method could be called upon in real time as a pulmonologist moves the pCLE probe through the lung and identifies a region of interest. The generated quantitative values can then be used to support the diagnosis of DPLD. Additionally, the suggested guiding capabilities of pCLE during cryobiopsy [33] can also benefit from the presented method’s ability to highlight DPLD-structures by offering further objective validation before sampling.

Since the algorithm used for segmentation is open source, its functions can easily be called from other script languages. As more data is collected, the training model’s segmentation capabilities can certainly be improved but also allows for the use of more complex methods. With deep learning approaches for example, structures could furthermore be fully automatically selected, segmented [34] before analysis with the presented method. This would further the methods real-time capability and offer attending physicians suggestions of interesting ROI rather than confirmation.

There are some limitations in this study. The primary factors are the absence of a histological comparison to pCLE snapshots from the same regions in the lungs resulting in some uncertainty as to whether pCLE can offer ‘optical biopsy’. Although the automatic segmentation’s performance was satisfactory on most snapshots a larger measurement set is required to assess its capabilities on different DPLDs and still unseen descriptors by the machine learning algorithm.

Given that the snapshots are taken from a very small fraction of the whole lung this could lead to a poor morphological consistency of the evaluation method [16]. However, this is mitigated already by the use of a guiding tool such as HRCT to select representative areas of diseased tissue. Variability can also be decreased by utilizing average parameter values generated from multiple snapshots of different representative areas for every patient. Since pCLE is an in-vivo imaging technique complementing bronchoscopy, it does not suffer the same increase of risk associated with invasive techniques such as biopsy and could be used to examine multiple sections. So far, procedures performed in multiple studies have shown no severe side effects other than minor bleeding in 3 out of 42 patients [13,30] making it ideal for longitudinal studies.

As the presented method evaluates 3D structures on 2D images, unattached structures such as macrophages in the alveolar space could potentially overlap and appear to be part of the structure of interest. Furthermore, the workflow relies on the operating pulmonologist to ensure a proper orientation of the probe relative to the area of interest. The first problem was partly mitigated in this study by training the machine learning method to distinguish background structures from structures of interest, but more data would likely be required to test the model’s generality. Future studies can further incorporate macrophages as a third segmentation class to expand on the analysis potential of the method. Consideration can also be taken by the performing pulmonologists if structures are overlapping or probe orientation needs adjustment when selecting snapshot. Validation cohorts would be required to confirm the clinical relevance of this method. In this initial study we have focused on the distinction between normal and DPLD alveoli due to the small sample size. A next step would be to establish respective value ranges for different types of pathologies aiming to further assess the presented methods’ diagnostic potential.

## Conclusion

In this study we presented a method for the quantitative evaluation of alveolar elastin structure using pCLE images. We demonstrated that quantifying structural properties of the alveoli elastin, such as thickness and connectivity, allows for the differentiation of DPLD and normal lung tissue. We furthermore presented a framework for a fully automated workflow that can be easily implemented into pCLE examinations. This can offer further assistance by providing quantitative values to pulmonologists for the diagnosis of DPLDs.

## Supporting information

S1 Data (ZIP)
