# Supplementary figures and images for: Automated evaluation of probe-based confocal laser endomicroscopy in the lung
Source: PLoS One. 2020 May 6;15(5):e0232847. doi: 10.1371/journal.pone.0232847 (PMC7202624; doi:10.1371/journal.pone.0232847)

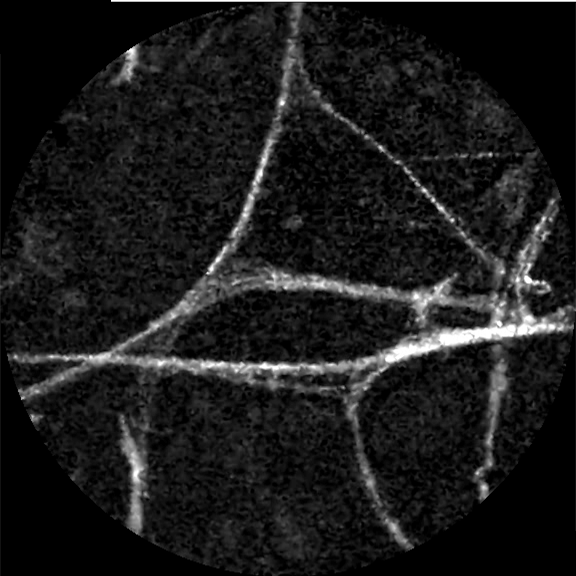

Supplement: S1 Data — (ZIP) [file pone.0232847.s001.zip › Data_for reproducing_methods/Data_for_final_structural_evaluation/comparative_set/_ABPA-Gruppe/Comp_set1/Original image.png]

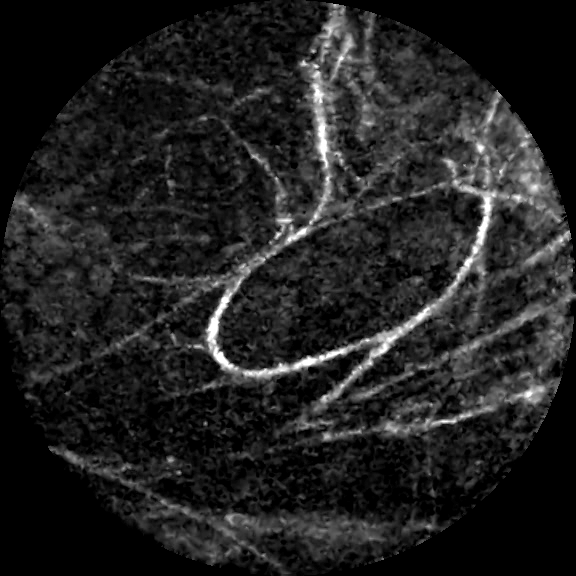

Supplement: S1 Data — (ZIP) [file pone.0232847.s001.zip › Data_for reproducing_methods/Data_for_final_structural_evaluation/comparative_set/_Entzundung-Gruppe/Comp_set2/Original image.png]

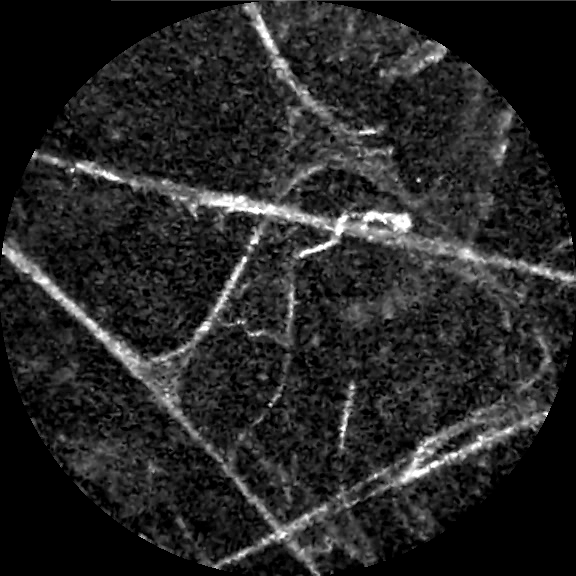

Supplement: S1 Data — (ZIP) [file pone.0232847.s001.zip › Data_for reproducing_methods/Data_for_final_structural_evaluation/comparative_set/_MammaCarcinom-Gruppe/Comp_set3/Original image.png]

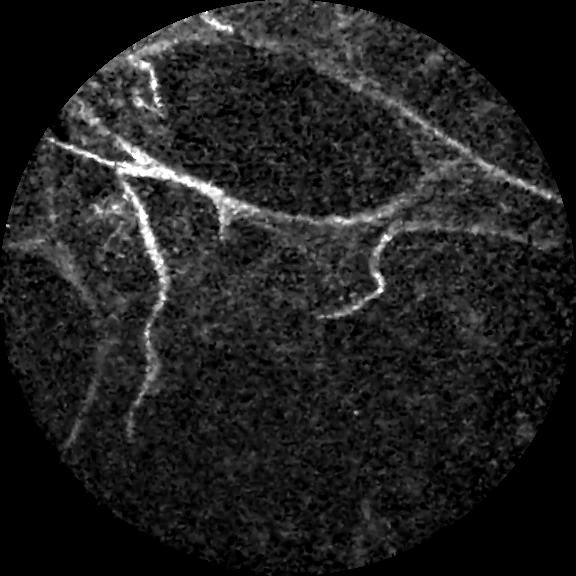

Supplement: S1 Data — (ZIP) [file pone.0232847.s001.zip › Data_for reproducing_methods/Data_for_final_structural_evaluation/comparative_set/_SARKOIDOSE-Gruppe/Comp_set4/Original image.png]

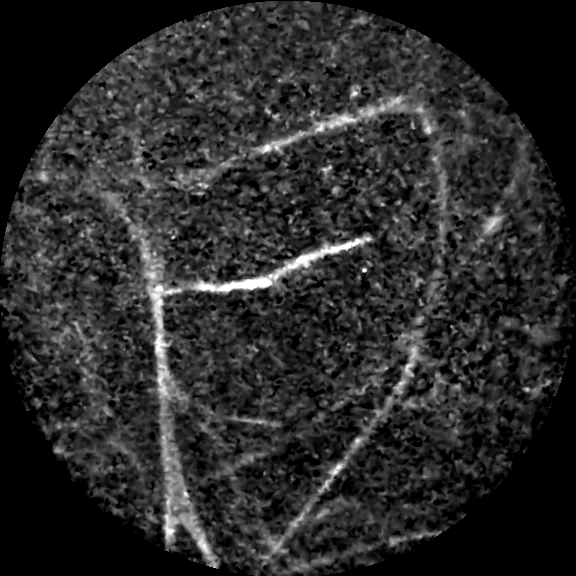

Supplement: S1 Data — (ZIP) [file pone.0232847.s001.zip › Data_for reproducing_methods/Data_for_final_structural_evaluation/comparative_set/_SARKOIDOSE-Gruppe/Comp_set5/Normal_Sarkoidose-Gruppe_GauBro39_1_.png]

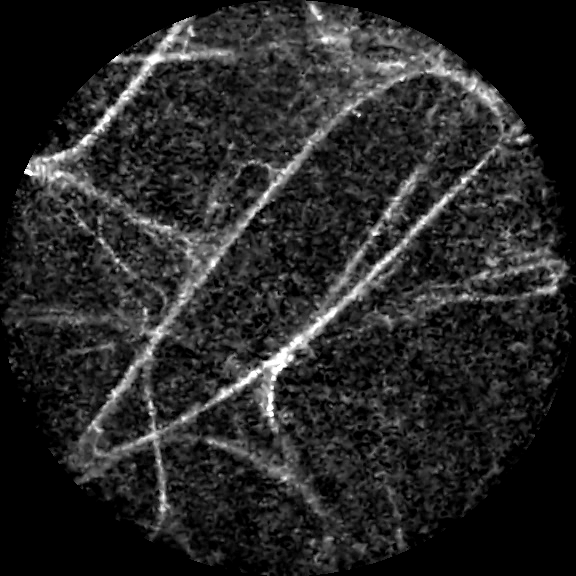

Supplement: S1 Data — (ZIP) [file pone.0232847.s001.zip › Data_for reproducing_methods/Data_for_final_structural_evaluation/comparative_set/_SARKOIDOSE-Gruppe/Comp_set6/Original image.png]

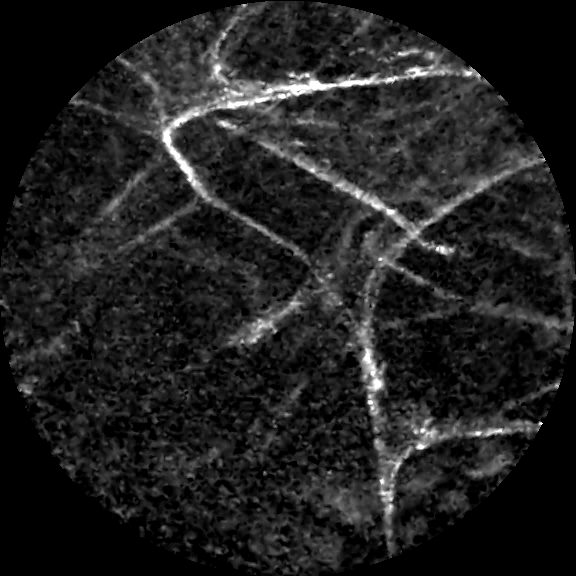

Supplement: S1 Data — (ZIP) [file pone.0232847.s001.zip › Data_for reproducing_methods/Data_for_final_structural_evaluation/comparative_set/_SARKOIDOSE-Gruppe/Comp_set7/Original image.png]

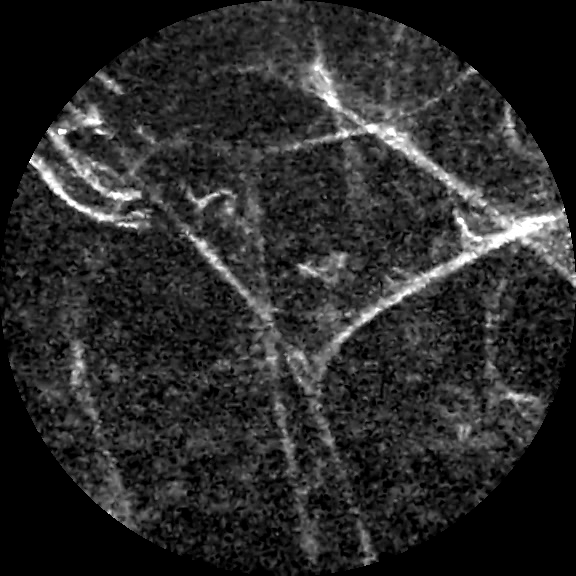

Supplement: S1 Data — (ZIP) [file pone.0232847.s001.zip › Data_for reproducing_methods/Data_for_final_structural_evaluation/comparative_set/_SARKOIDOSE-Gruppe/Comp_set8/Original image.png]

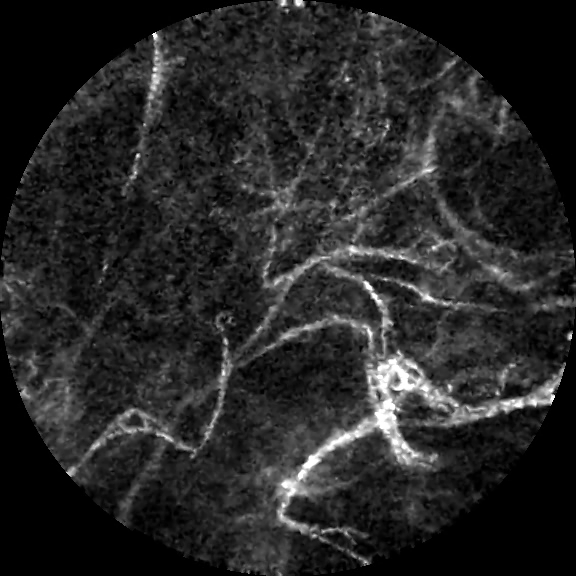

Supplement: S1 Data — (ZIP) [file pone.0232847.s001.zip › Data_for reproducing_methods/Data_for_final_structural_evaluation/COP-Gruppe/COP_10/Original image.png]

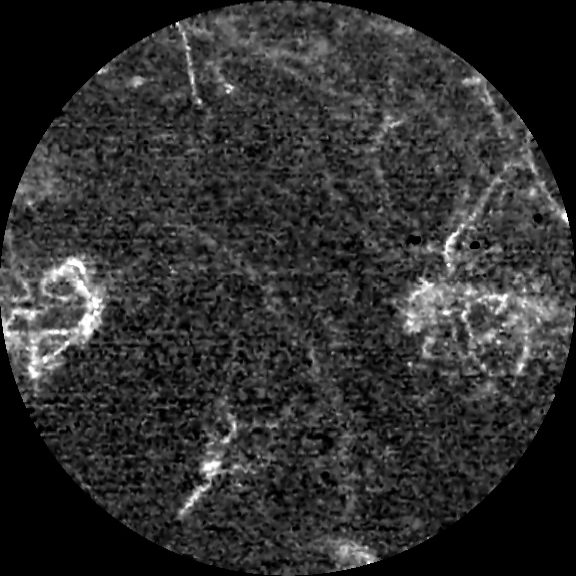

Supplement: S1 Data — (ZIP) [file pone.0232847.s001.zip › Data_for reproducing_methods/Data_for_final_structural_evaluation/COP-Gruppe/COP_11/COP-Gruppe_GauOP3_002-2015_Second26.png]

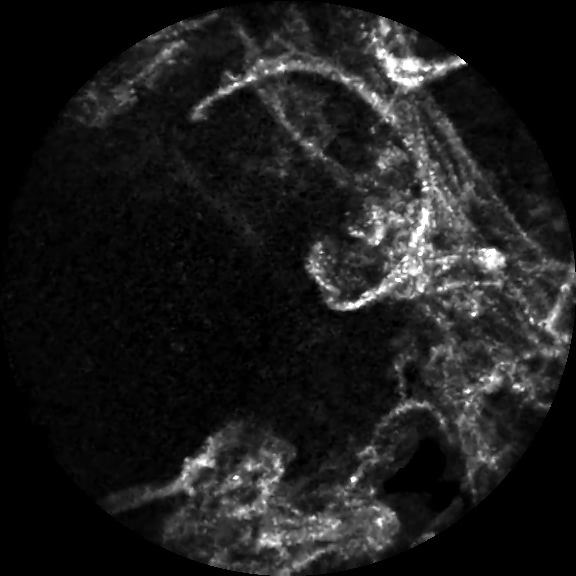

Supplement: S1 Data — (ZIP) [file pone.0232847.s001.zip › Data_for reproducing_methods/Data_for_final_structural_evaluation/COP-Gruppe/COP_1/Original image.png]

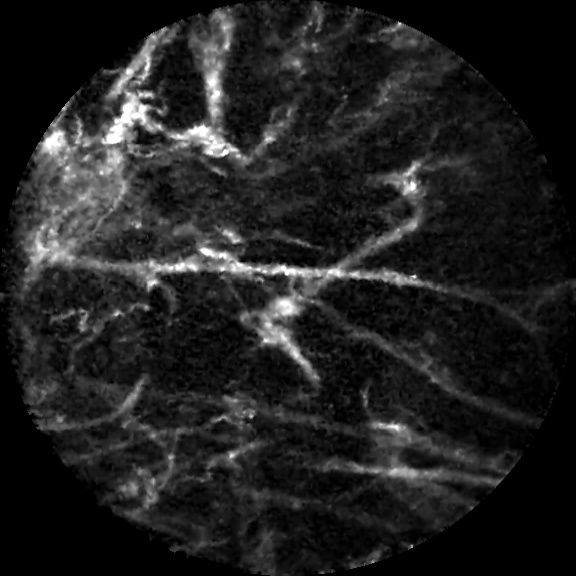

Supplement: S1 Data — (ZIP) [file pone.0232847.s001.zip › Data_for reproducing_methods/Data_for_final_structural_evaluation/COP-Gruppe/COP_2/Original image.png]

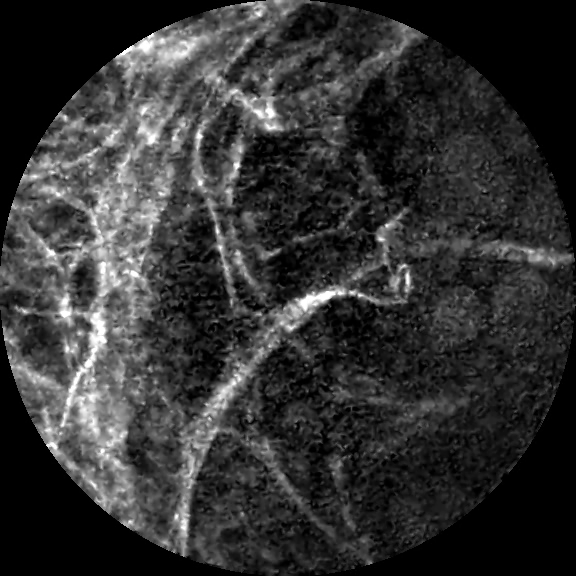

Supplement: S1 Data — (ZIP) [file pone.0232847.s001.zip › Data_for reproducing_methods/Data_for_final_structural_evaluation/COP-Gruppe/COP_3/Original image.png]

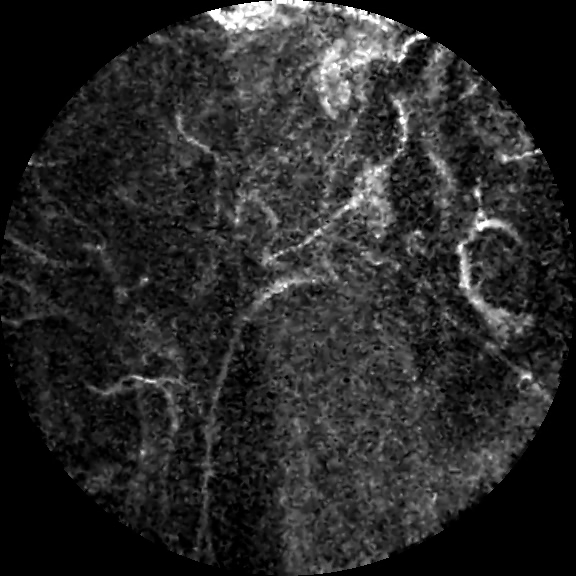

Supplement: S1 Data — (ZIP) [file pone.0232847.s001.zip › Data_for reproducing_methods/Data_for_final_structural_evaluation/COP-Gruppe/COP_4/Original image.png]

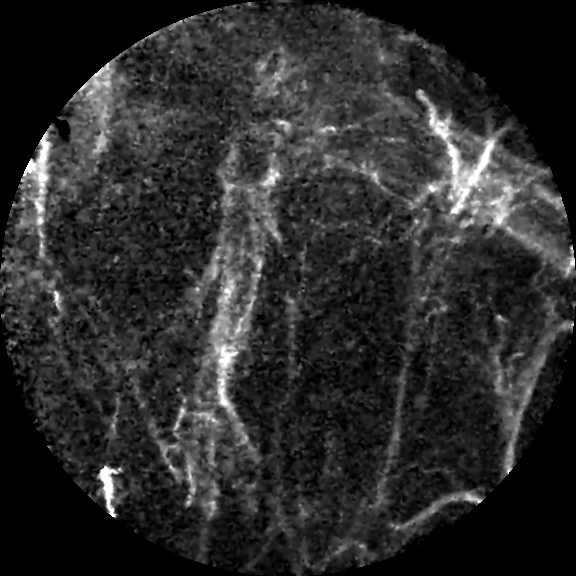

Supplement: S1 Data — (ZIP) [file pone.0232847.s001.zip › Data_for reproducing_methods/Data_for_final_structural_evaluation/COP-Gruppe/COP_5/Original image.png]

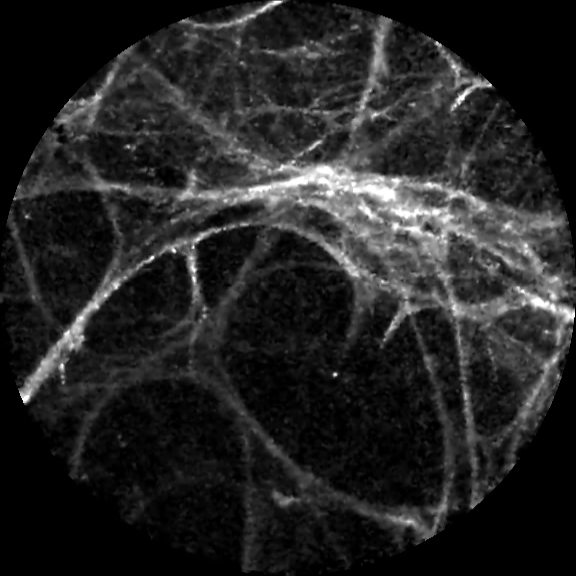

Supplement: S1 Data — (ZIP) [file pone.0232847.s001.zip › Data_for reproducing_methods/Data_for_final_structural_evaluation/COP-Gruppe/COP_6/Original image.png]

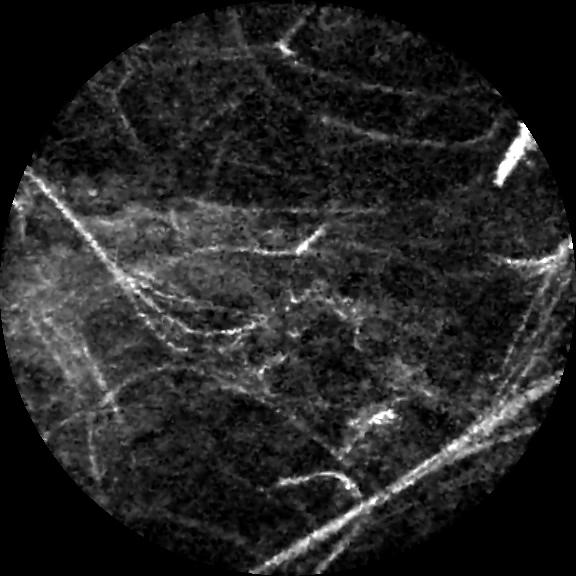

Supplement: S1 Data — (ZIP) [file pone.0232847.s001.zip › Data_for reproducing_methods/Data_for_final_structural_evaluation/COP-Gruppe/COP_7/Original image.png]

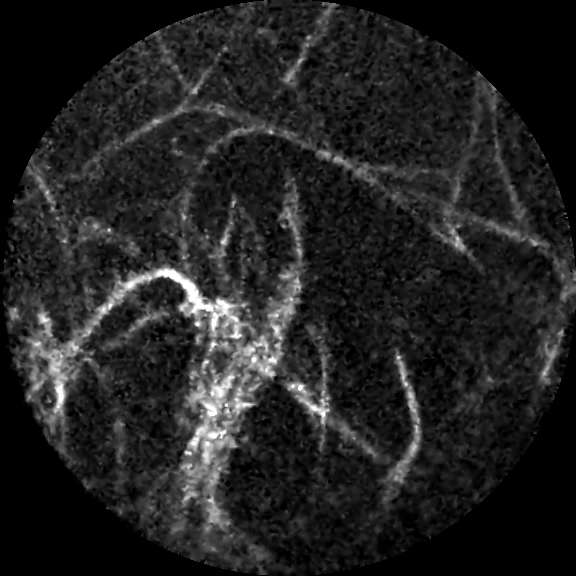

Supplement: S1 Data — (ZIP) [file pone.0232847.s001.zip › Data_for reproducing_methods/Data_for_final_structural_evaluation/COP-Gruppe/COP_8/Original image.png]

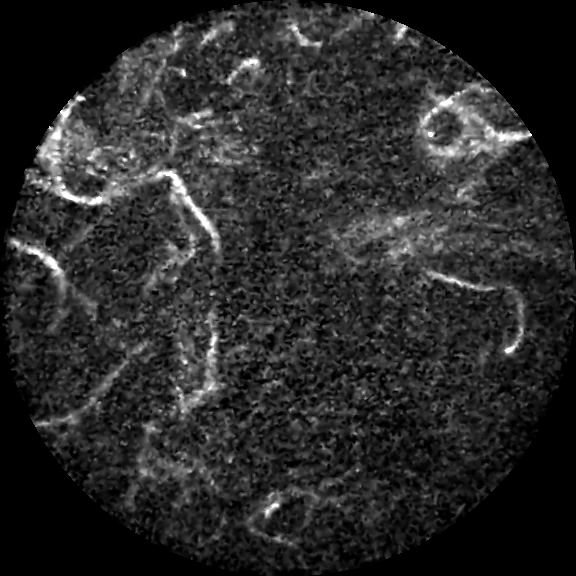

Supplement: S1 Data — (ZIP) [file pone.0232847.s001.zip › Data_for reproducing_methods/Data_for_final_structural_evaluation/COP-Gruppe/COP_9/COP-Gruppe_GauBro54_003-2016_Second20.png]

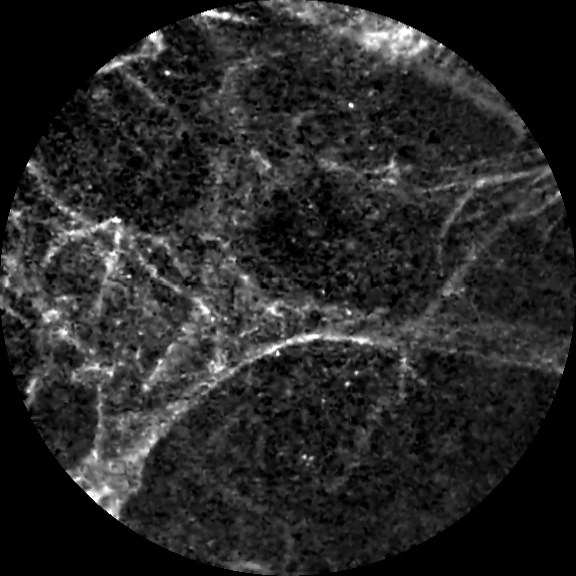

Supplement: S1 Data — (ZIP) [file pone.0232847.s001.zip › Data_for reproducing_methods/Data_for_final_structural_evaluation/HP-GRUPPE/HP_1/Original image.png]

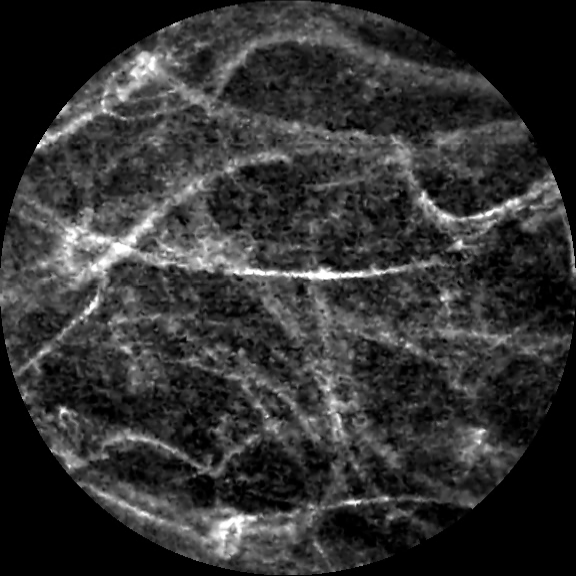

Supplement: S1 Data — (ZIP) [file pone.0232847.s001.zip › Data_for reproducing_methods/Data_for_final_structural_evaluation/HP-GRUPPE/HP_2/Original image.png]

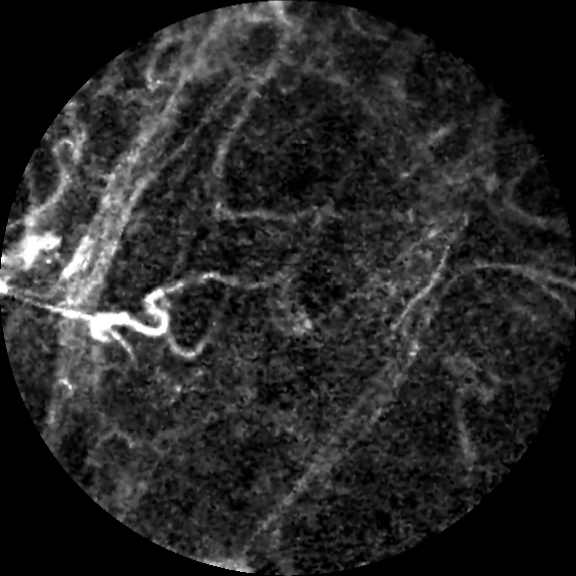

Supplement: S1 Data — (ZIP) [file pone.0232847.s001.zip › Data_for reproducing_methods/Data_for_final_structural_evaluation/HP-GRUPPE/HP_3/Original image.png]

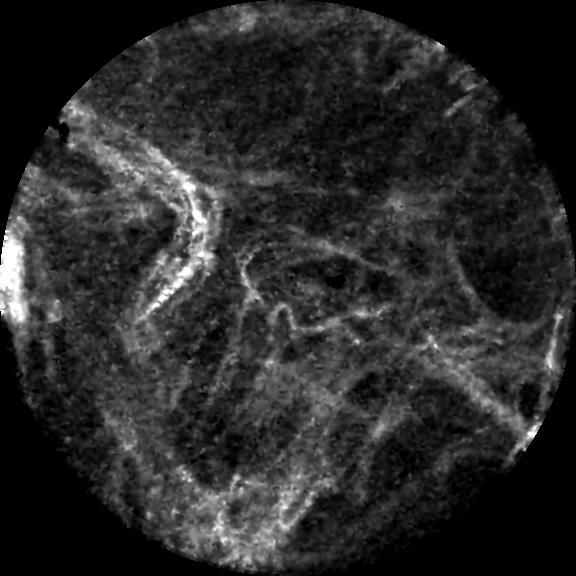

Supplement: S1 Data — (ZIP) [file pone.0232847.s001.zip › Data_for reproducing_methods/Data_for_final_structural_evaluation/HP-GRUPPE/HP_4/Original image.png]

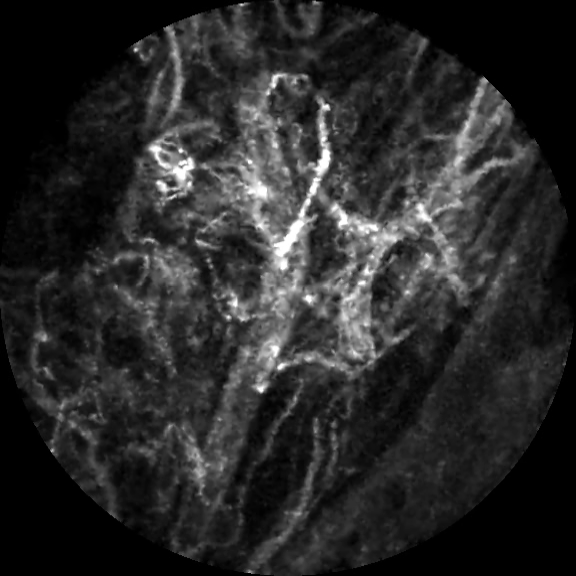

Supplement: S1 Data — (ZIP) [file pone.0232847.s001.zip › Data_for reproducing_methods/Data_for_final_structural_evaluation/HP-GRUPPE/HP_5/Original image.png]

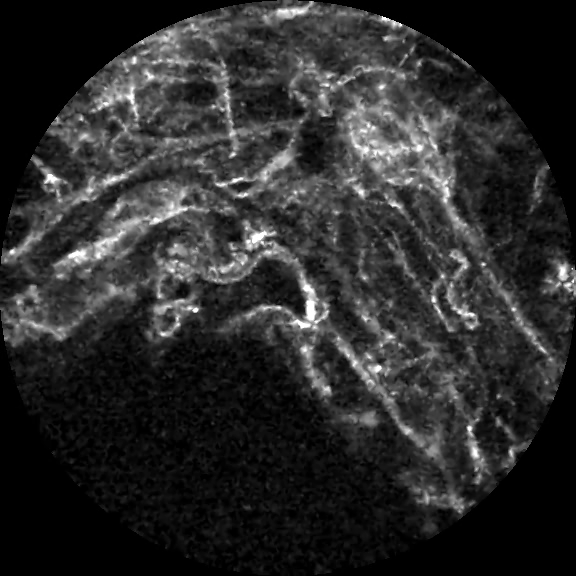

Supplement: S1 Data — (ZIP) [file pone.0232847.s001.zip › Data_for reproducing_methods/Data_for_final_structural_evaluation/HP-GRUPPE/HP_6/Original image.png]

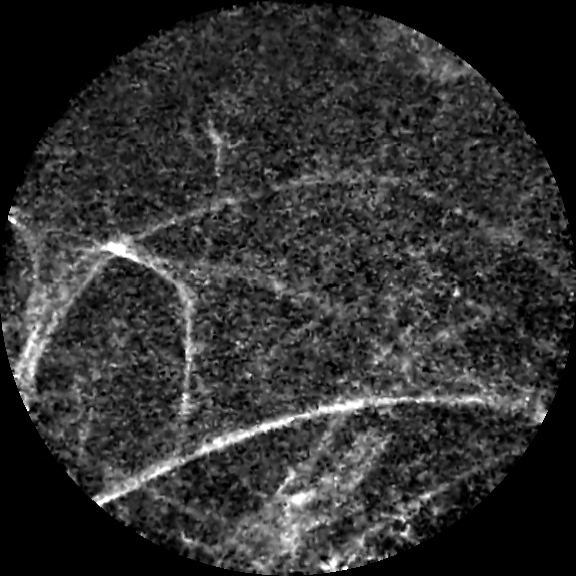

Supplement: S1 Data — (ZIP) [file pone.0232847.s001.zip › Data_for reproducing_methods/Data_for_final_structural_evaluation/HP-GRUPPE/HP_7/Original image.png]

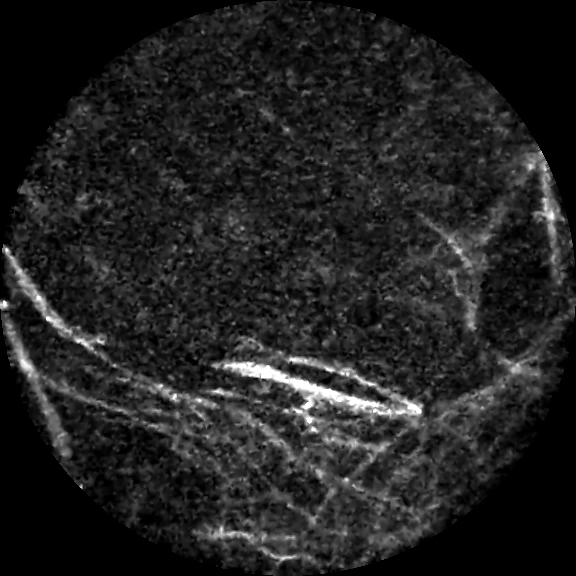

Supplement: S1 Data — (ZIP) [file pone.0232847.s001.zip › Data_for reproducing_methods/Data_for_final_structural_evaluation/IPF-Gruppe/IPF_10/Original image.png]

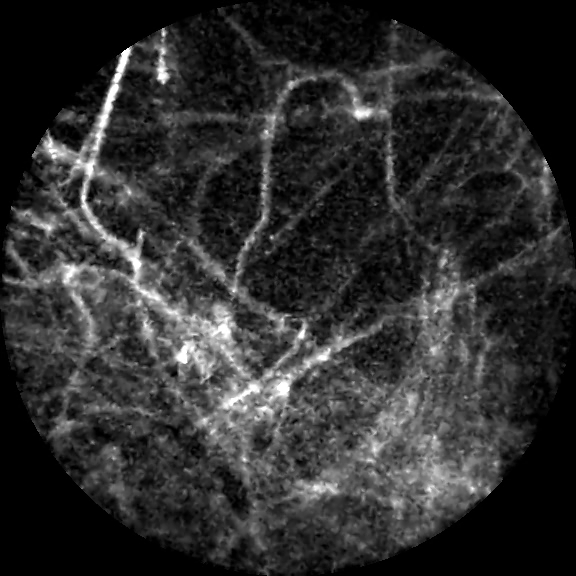

Supplement: S1 Data — (ZIP) [file pone.0232847.s001.zip › Data_for reproducing_methods/Data_for_final_structural_evaluation/IPF-Gruppe/IPF_11/Original image.png]

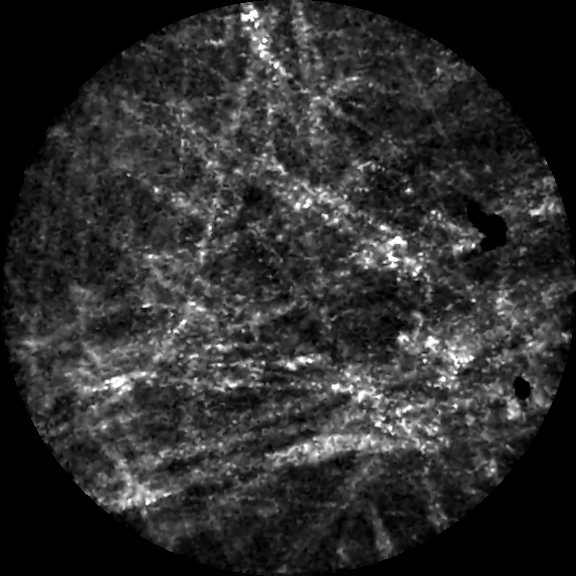

Supplement: S1 Data — (ZIP) [file pone.0232847.s001.zip › Data_for reproducing_methods/Data_for_final_structural_evaluation/IPF-Gruppe/IPF_1/Original image.png]

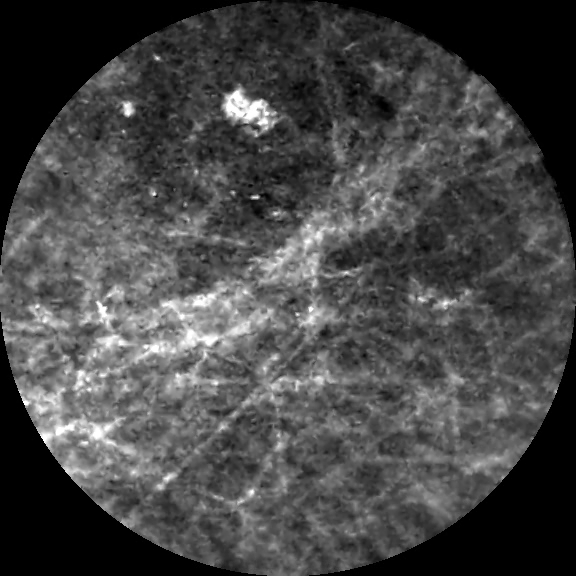

Supplement: S1 Data — (ZIP) [file pone.0232847.s001.zip › Data_for reproducing_methods/Data_for_final_structural_evaluation/IPF-Gruppe/IPF_2/Original image.png]

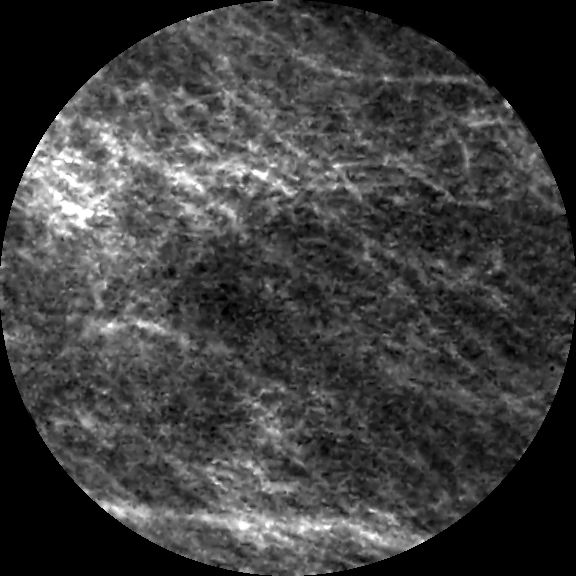

Supplement: S1 Data — (ZIP) [file pone.0232847.s001.zip › Data_for reproducing_methods/Data_for_final_structural_evaluation/IPF-Gruppe/IPF_3/Original image.png]

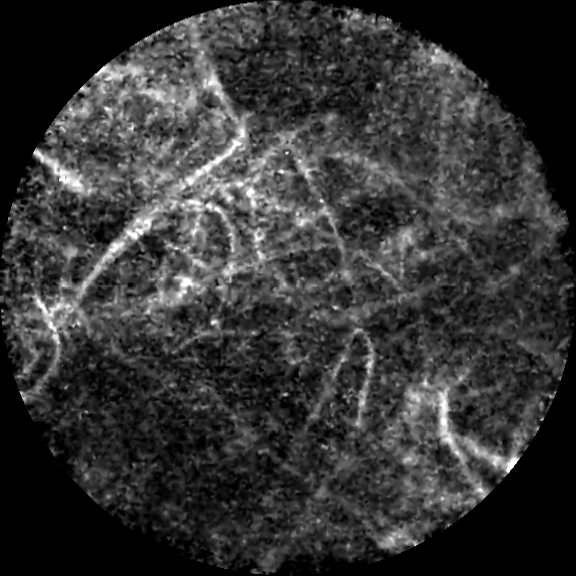

Supplement: S1 Data — (ZIP) [file pone.0232847.s001.zip › Data_for reproducing_methods/Data_for_final_structural_evaluation/IPF-Gruppe/IPF_4/Original image.png]

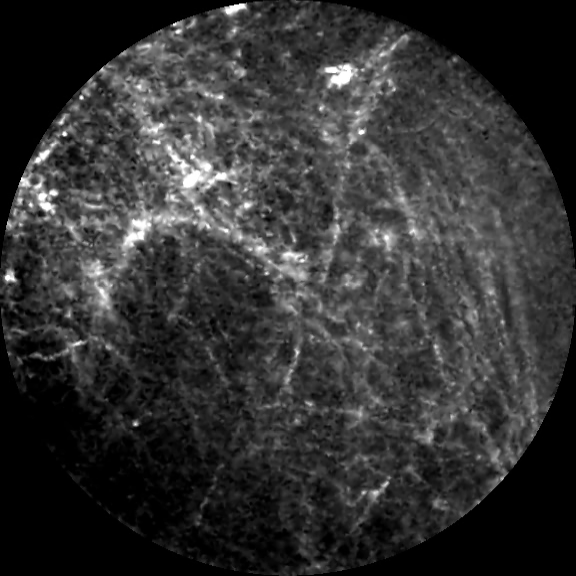

Supplement: S1 Data — (ZIP) [file pone.0232847.s001.zip › Data_for reproducing_methods/Data_for_final_structural_evaluation/IPF-Gruppe/IPF_5/Original image.png]

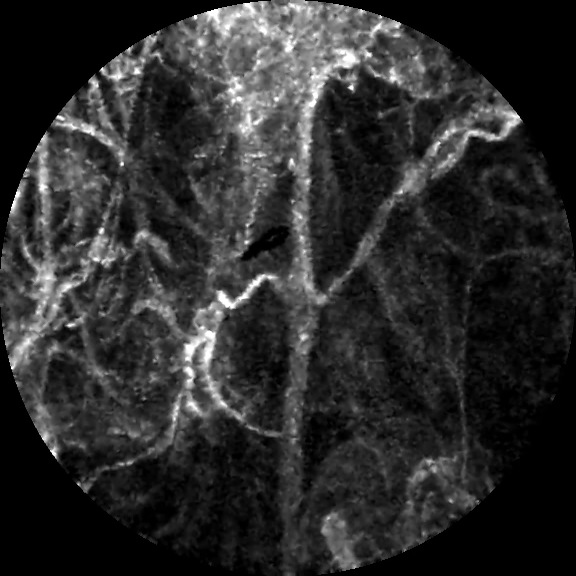

Supplement: S1 Data — (ZIP) [file pone.0232847.s001.zip › Data_for reproducing_methods/Data_for_final_structural_evaluation/IPF-Gruppe/IPF_6/Original image.png]

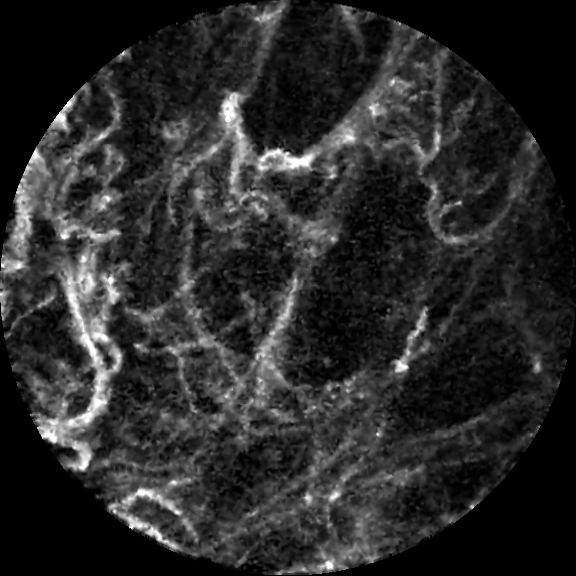

Supplement: S1 Data — (ZIP) [file pone.0232847.s001.zip › Data_for reproducing_methods/Data_for_final_structural_evaluation/IPF-Gruppe/IPF_7/Original image.png]

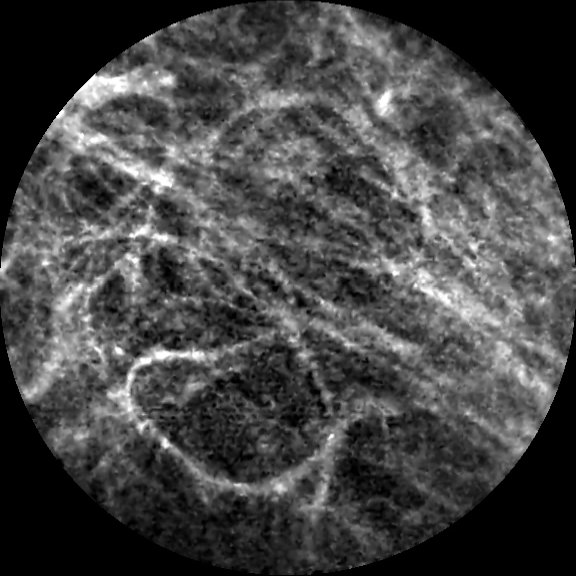

Supplement: S1 Data — (ZIP) [file pone.0232847.s001.zip › Data_for reproducing_methods/Data_for_final_structural_evaluation/IPF-Gruppe/IPF_8/Original image.png]

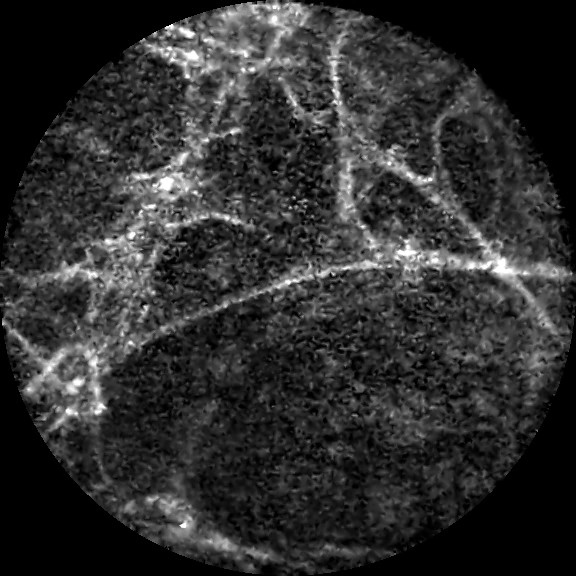

Supplement: S1 Data — (ZIP) [file pone.0232847.s001.zip › Data_for reproducing_methods/Data_for_final_structural_evaluation/IPF-Gruppe/IPF_9/Original image.png]

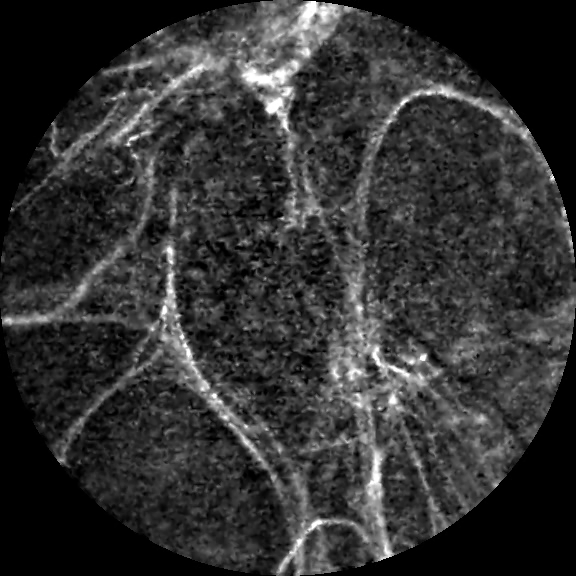

Supplement: S1 Data — (ZIP) [file pone.0232847.s001.zip › Data_for reproducing_methods/Data_for_final_structural_evaluation/NSIP-Gruppe/NSIP_1/Original image.png]

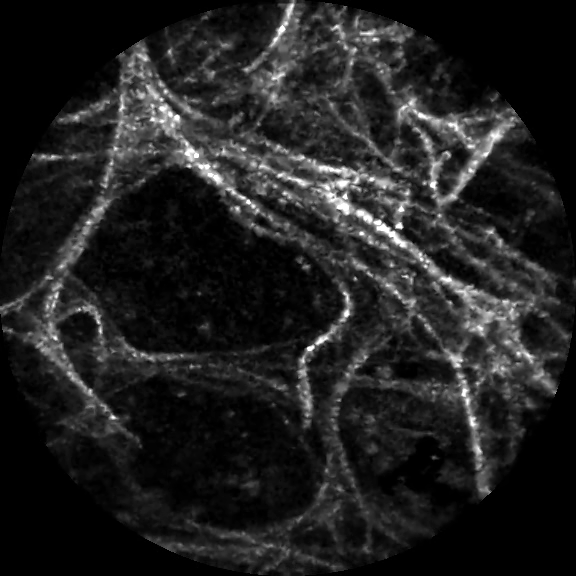

Supplement: S1 Data — (ZIP) [file pone.0232847.s001.zip › Data_for reproducing_methods/Data_for_final_structural_evaluation/NSIP-Gruppe/NSIP_2/Original image.png]

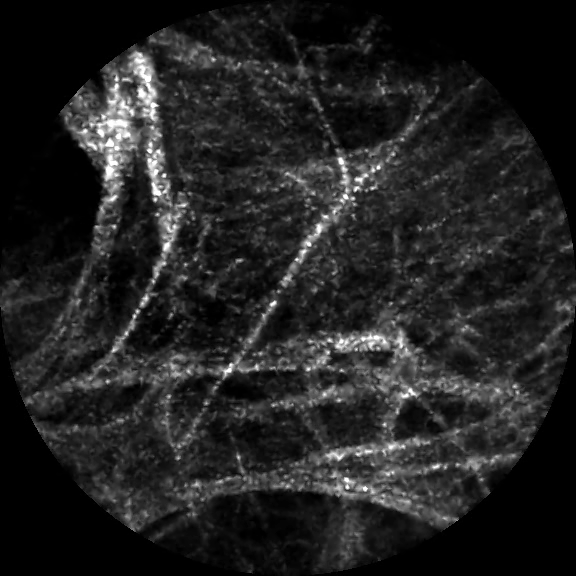

Supplement: S1 Data — (ZIP) [file pone.0232847.s001.zip › Data_for reproducing_methods/Data_for_final_structural_evaluation/NSIP-Gruppe/NSIP_3/Original image.png]

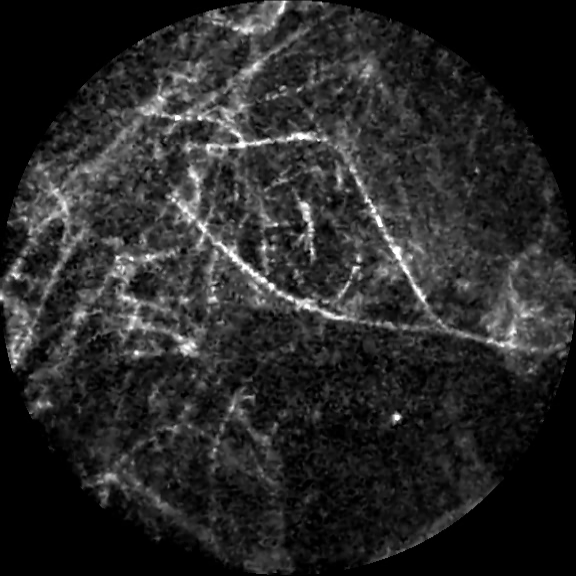

Supplement: S1 Data — (ZIP) [file pone.0232847.s001.zip › Data_for reproducing_methods/Data_for_final_structural_evaluation/NSIP-Gruppe/NSIP_4/Original image.png]

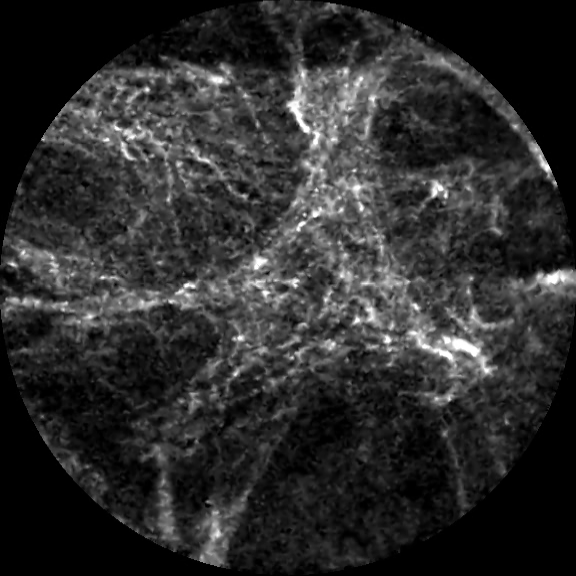

Supplement: S1 Data — (ZIP) [file pone.0232847.s001.zip › Data_for reproducing_methods/Data_for_final_structural_evaluation/NSIP-Gruppe/NSIP_5/Original image.png]

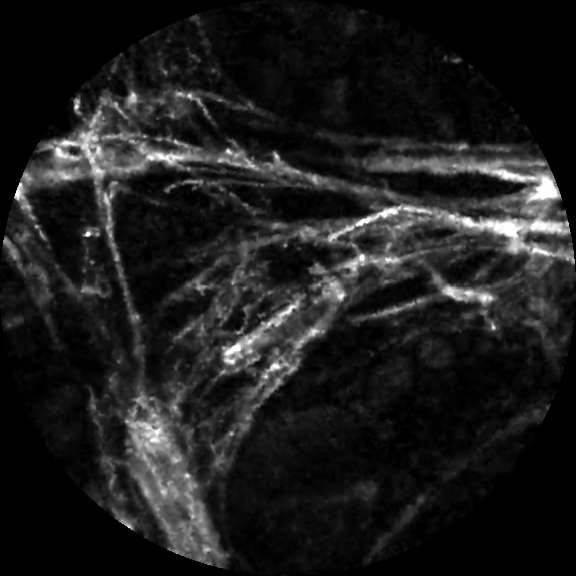

Supplement: S1 Data — (ZIP) [file pone.0232847.s001.zip › Data_for reproducing_methods/Data_for_final_structural_evaluation/NSIP-Gruppe/NSIP_6/Original image.png]

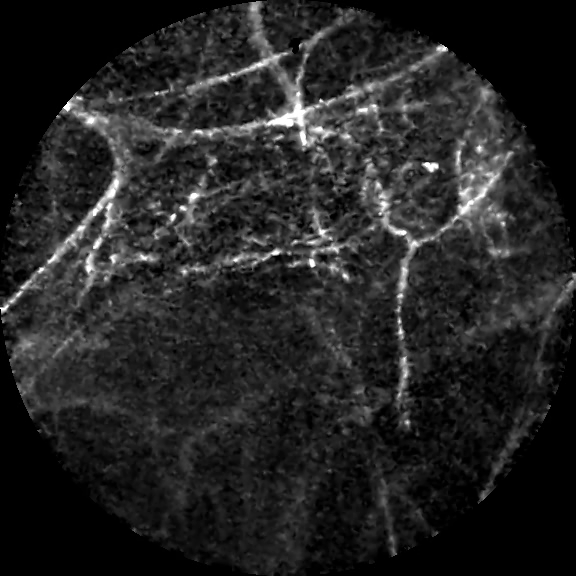

Supplement: S1 Data — (ZIP) [file pone.0232847.s001.zip › Data_for reproducing_methods/Data_for_final_structural_evaluation/NSIP-Gruppe/NSIP_7/Original image.png]

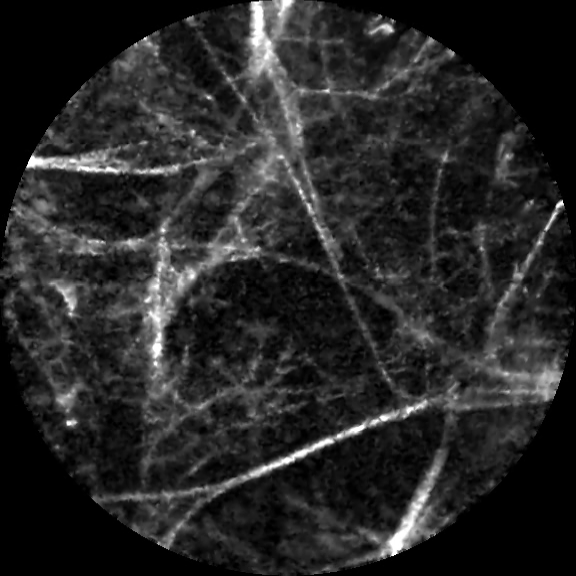

Supplement: S1 Data — (ZIP) [file pone.0232847.s001.zip › Data_for reproducing_methods/Data_for_final_structural_evaluation/NSIP-Gruppe/NSIP_8/Original image.png]

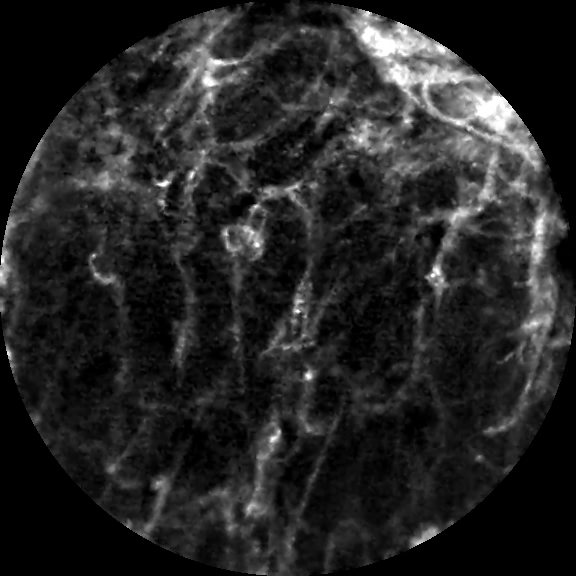

Supplement: S1 Data — (ZIP) [file pone.0232847.s001.zip › Data_for reproducing_methods/Training_data_with_ROI/Test_images/IPF_16.png]

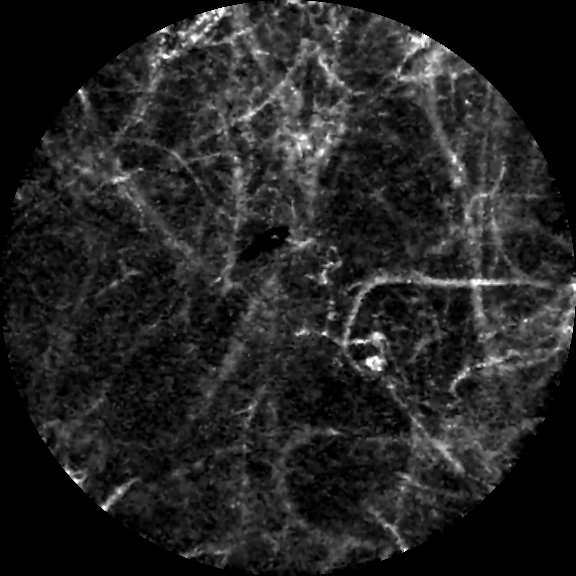

Supplement: S1 Data — (ZIP) [file pone.0232847.s001.zip › Data_for reproducing_methods/Training_data_with_ROI/Test_images/IPF_17.png]

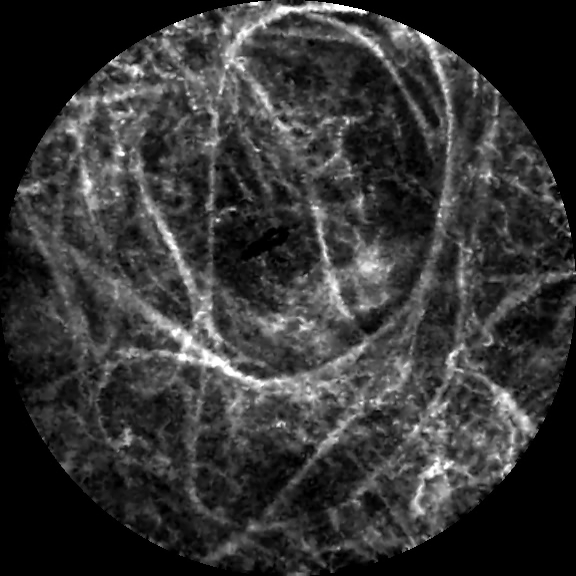

Supplement: S1 Data — (ZIP) [file pone.0232847.s001.zip › Data_for reproducing_methods/Training_data_with_ROI/Training_set/Training_images/IPF_10.png]

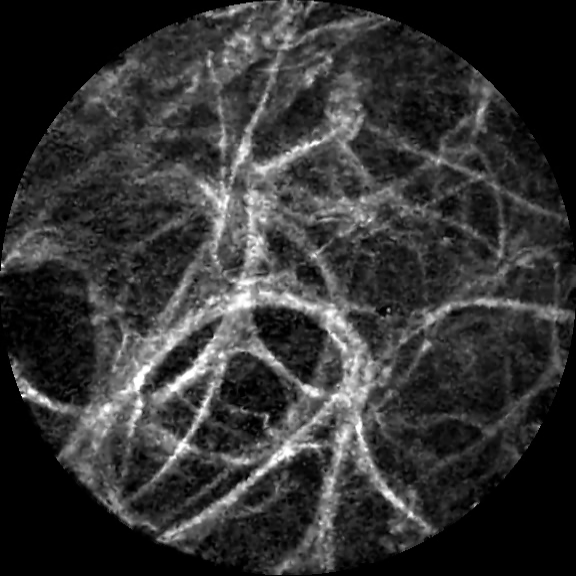

Supplement: S1 Data — (ZIP) [file pone.0232847.s001.zip › Data_for reproducing_methods/Training_data_with_ROI/Training_set/Training_images/IPF_12.png]

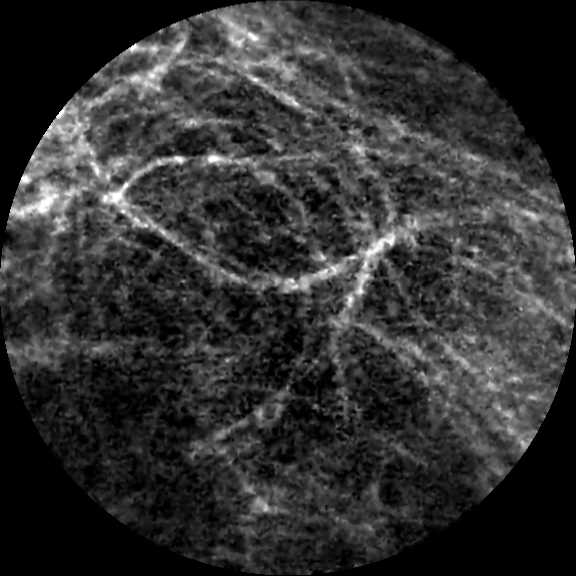

Supplement: S1 Data — (ZIP) [file pone.0232847.s001.zip › Data_for reproducing_methods/Training_data_with_ROI/Training_set/Training_images/IPF_14.png]

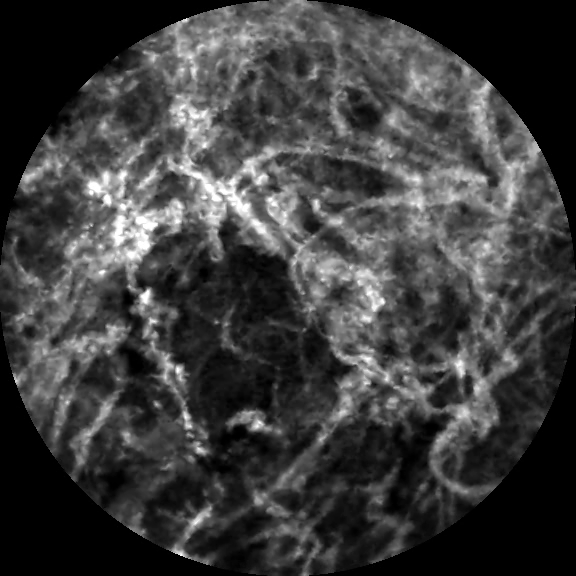

Supplement: S1 Data — (ZIP) [file pone.0232847.s001.zip › Data_for reproducing_methods/Training_data_with_ROI/Training_set/Training_images/IPF_2.png]

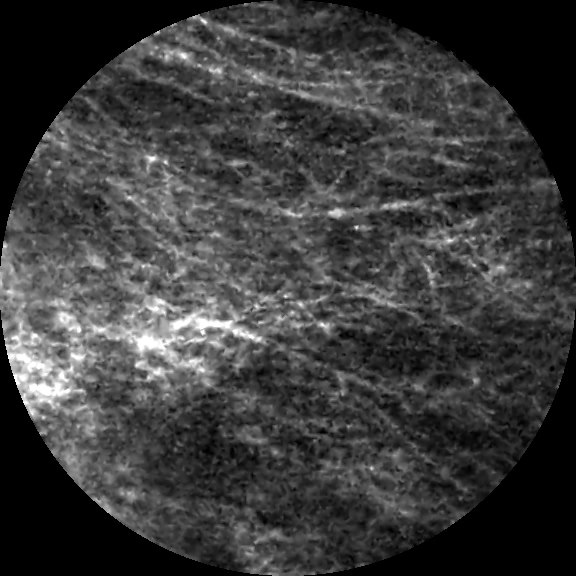

Supplement: S1 Data — (ZIP) [file pone.0232847.s001.zip › Data_for reproducing_methods/Training_data_with_ROI/Training_set/Training_images/IPF_4.png]

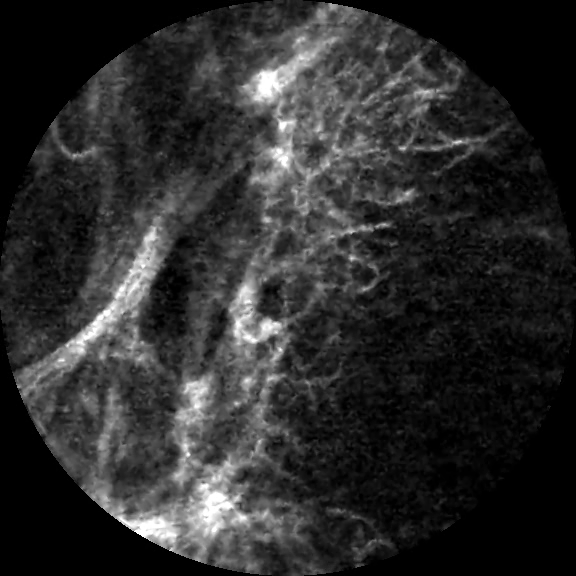

Supplement: S1 Data — (ZIP) [file pone.0232847.s001.zip › Data_for reproducing_methods/Training_data_with_ROI/Training_set/Training_images/IPF_5.png]

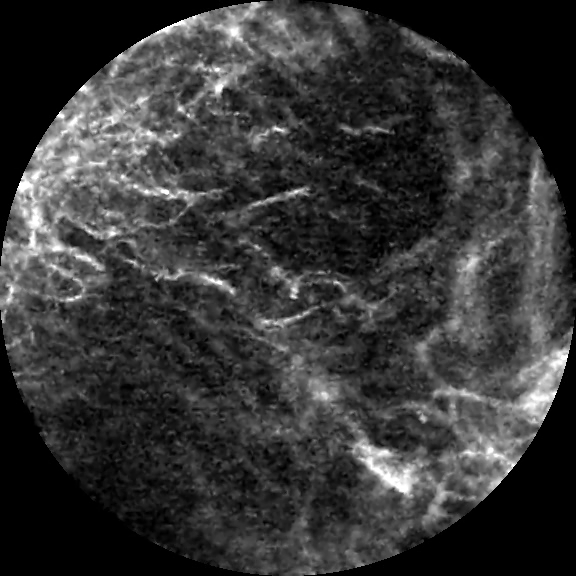

Supplement: S1 Data — (ZIP) [file pone.0232847.s001.zip › Data_for reproducing_methods/Training_data_with_ROI/Training_set/Training_images/IPF_6.png]

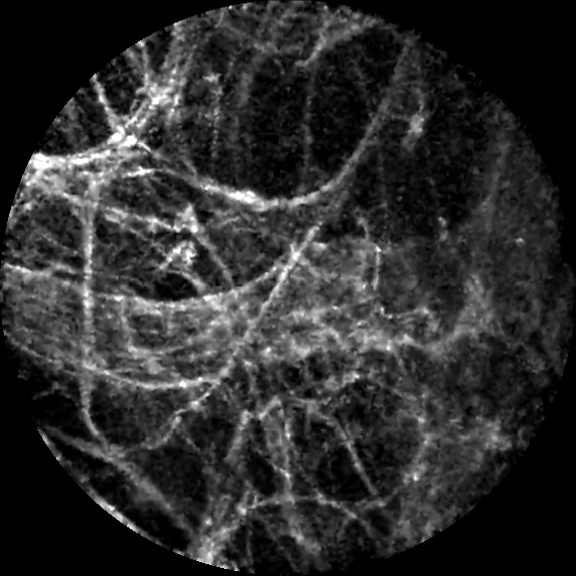

Supplement: S1 Data — (ZIP) [file pone.0232847.s001.zip › Data_for reproducing_methods/Training_data_with_ROI/Training_set/Training_images/IPF_7.png]

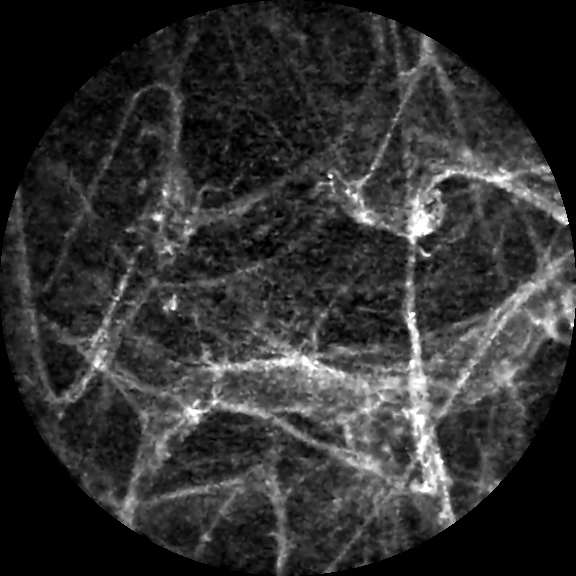

Supplement: S1 Data — (ZIP) [file pone.0232847.s001.zip › Data_for reproducing_methods/Training_data_with_ROI/Training_set/Training_images/NSIP_2.png]

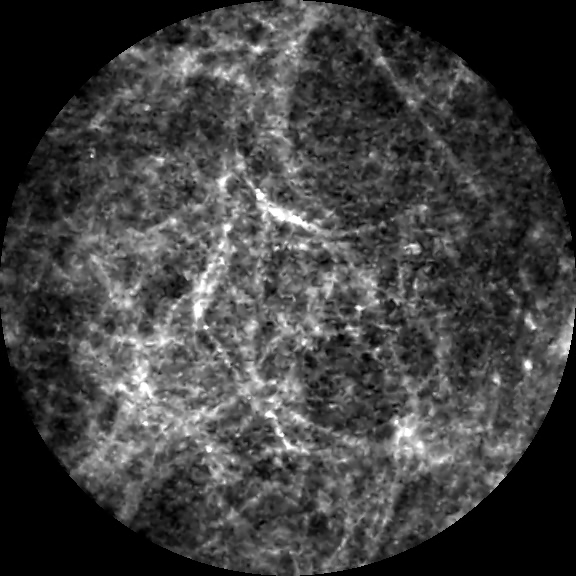

Supplement: S1 Data — (ZIP) [file pone.0232847.s001.zip › Data_for reproducing_methods/Training_data_with_ROI/Training_set/Training_images/NSIP_3.png]

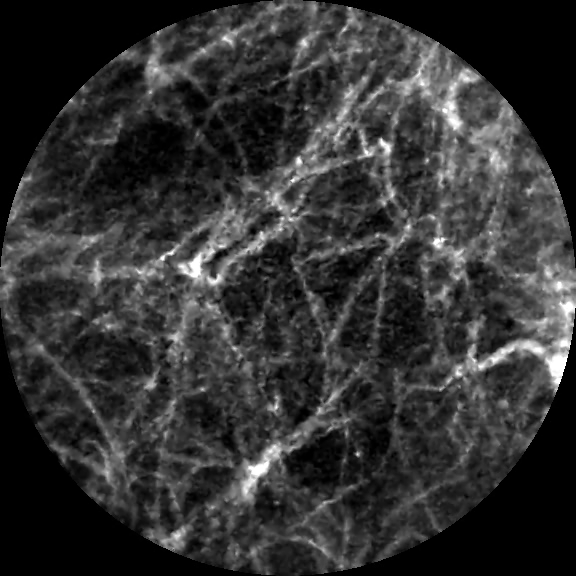

Supplement: S1 Data — (ZIP) [file pone.0232847.s001.zip › Data_for reproducing_methods/Training_data_with_ROI/Training_set/Training_images/NSIP_4.png]

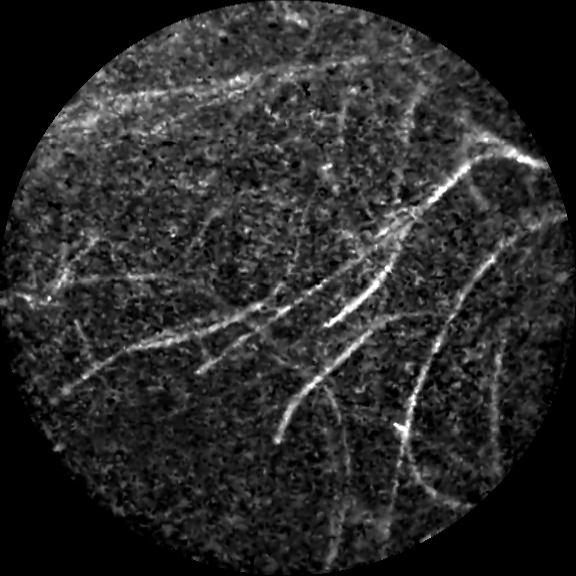

Supplement: S1 Data — (ZIP) [file pone.0232847.s001.zip › Data_for reproducing_methods/Training_data_with_ROI/Training_set/Training_images/Sarkoidose_2.png]
